# Global Poultry Feed Protein Sources: Trends, Challenges, and Sustainability Perspectives Using FAOSTAT Data

**DOI:** 10.1111/jpn.70104

**Published:** 2026-08-10

**Authors:** Sajjad Hassan Qadeer

**Affiliations:** ^1^ Institute of Physiology and Nutrition Hungarian University of Agriculture and Life Sciences (MATE) Kaposvár Hungary

**Keywords:** alternative proteins, feed security, insect meal, poultry nutrition, sustainability, systematic review

## Abstract

The global poultry industry is one of the fastest‐growing livestock sectors, with worldwide chicken stocks exceeding 32 billion heads and meat production surpassing 144 million tonnes in 2024 (FAOSTAT, 2026). Conventional protein sources, particularly soybean meal (SBM) and fishmeal, underpin most commercial poultry diets but face mounting challenges related to environmental sustainability, supply‐chain volatility, and land‐use pressures. This systematic review evaluates global trends in poultry feed protein sources using FAOSTAT production and trade data (2000–2024), alongside evidence from 57 peer‐reviewed publications identified through a PRISMA‐compliant, multi‐database search. A structured literature search was conducted across Web of Science (WoS), Scopus, and PubMed using consistent search logic combining poultry, protein‐source, and sustainability/nutrition/feed terms. Searches yielded 122 (WoS), 5920 (Scopus), and 1827 (PubMed) records (7869 total; 6215 unique after removing ineligible document types and DOI‐matched duplicates). The qualitative synthesis presented here is based on the original WoS‐driven screening pipeline (57 included studies); the wider Scopus/PubMed pool was used as a supplementary coverage check rather than fully re‐screened, a limitation discussed in Section 4.6. FAOSTAT data sets on chicken stocks, meat production, soybean production, and soybean trade were analysed for 2000–2024. Global chicken stocks grew from ~14.8 billion head in 2000 to ~32.2 billion in 2024, while global soybean production increased from 161 Mt to 418 Mt over the same period. Alternative protein sources—particularly black soldier fly larvae (BSFL), mealworms, single‐cell proteins (SCP), microalgae, and co‐products such as distillers' dried grains with solubles (DDGS)—demonstrate high potential as partial or full substitutes for SBM and fishmeal in poultry diets. However, challenges including regulatory restrictions, inconsistent nutritional profiles, anti‐nutritional factors (ANFs), and production scalability remain significant barriers. Transitioning to diversified, locally sourced, and circular‐economy‐based protein ingredients is essential to ensure long‐term sustainability of the global poultry industry. Policy harmonisation, technological innovation, and consumer acceptance research are critical priority areas for future work.

## 1 Introduction

The global poultry industry occupies a central position in the world food system, supplying affordable animal protein across all income levels. Global chicken stocks have more than doubled since 2000, driven by rising per‐capita income in developing countries, rapid urbanisation, and the comparatively low environmental footprint of poultry relative to beef and pork (Selaledi et al. 2018; Wiedemann et al. 2013). Detailed production figures are presented in Section 3.1 rather than repeated here.

Feed costs typically account for 60%–70% of total poultry production costs, with protein ingredients—primarily soybean meal (SBM) and fishmeal—representing the single largest nutritional expense. Global soybean production has nearly tripled since 2000 (FAOSTAT, 2026), an expansion that has required substantial agricultural land conversion, particularly in South America. Deforestation associated with soybean cultivation, greenhouse gas (GHG) emissions, water‐use intensity, and geopolitical supply‐chain risks increasingly threaten the long‐term viability of SBM as the cornerstone of poultry nutrition (Nyameasem et al. 2026; Wiedemann et al. 2013).

Fishmeal, which provides highly digestible protein and essential amino acids including methionine and lysine, faces the additional problem of declining marine fish stocks and rising global demand from aquaculture. Prices have become increasingly volatile, and the long‐term availability of fishmeal for terrestrial livestock, including poultry, is uncertain (Tacon and Metian 2009).

Against this backdrop, a global research effort has been directed towards identifying and validating alternative protein sources for poultry. Insect meals—particularly from black soldier fly larvae (BSFL, Hermetia illucens) and yellow mealworm (Tenebrio molitor)—have attracted intense scientific interest because of their high protein content (40%–60% crude protein, CP), amino acid profiles, circular‐economy credentials, and ability to be reared on organic waste streams (Zaid et al. 2023; de Souza‐Vilela et al. 2019; Al‐Qazzaz and Ismail 2016). Single‐cell proteins (SCP) derived from yeast, bacteria, or algae offer additional possibilities for decoupling protein production from arable land use (García‐Ruiz and Durán‐Meléndez 2022). Co‐products from biofuel and starch industries such as distillers' dried grains with solubles (DDGS) and canola meal provide cost‐effective alternatives that are readily available in regions where the relevant processing infrastructure already exists (Burton et al. 2013; Lim et al. 2008).

### 1.1 Novelty and Research Gap

Several narrative and systematic reviews have previously addressed individual alternative protein sources for poultry—for example, insect meals (Sajid et al. 2023; Bellezza Oddon et al. 2025) or full‐fat soybean processing (Toomer et al. 2024)—typically in isolation from one another and without reference to macro‐level production and trade data. The present review differs in three respects: (1) it is, to our knowledge, the first to integrate FAOSTAT global production and trade statistics directly with a PRISMA‐compliant qualitative synthesis of protein‐source literature, allowing sustainability claims to be grounded in quantified global supply trends rather than qualitative assertion alone; (2) it evaluates conventional and emerging protein sources side‐by‐side within a single comparative framework (see Section 3.3) rather than as separate single‐ingredient reviews; and (3) it explicitly addresses cross‐database search coverage (Section 2.2), a methodological transparency step often absent from single‐database narrative reviews in this field.

The specific research gap addressed is the disconnect between techno‐nutritional feasibility studies (which show alternative proteins can replace SBM/fishmeal at the trial level) and global supply‐chain sustainability data (which show continued near‐total dependence on soybean and fishmeal at the macro level). Bridging these two literatures is the primary contribution of this review.

### 1.2 Objectives

This systematic review, conducted in accordance with PRISMA guidelines, synthesises evidence from 57 peer‐reviewed publications and integrates FAOSTAT production and trade data (2000–2024) to: (1) quantify global trends in poultry production and primary protein‐source availability; (2) compare the nutritional characteristics and sustainability profiles of major and emerging protein sources for poultry; (3) identify key challenges and research gaps; and (4) provide evidence‐informed recommendations for future protein‐sourcing strategies.

## 2 Methods

### 2.1 Protocol and Registration

This review was conducted in accordance with the Preferred Reporting Items for Systematic Reviews and Meta‐Analyses (PRISMA) 2020 guidelines (Page 2021). The review was not pre‐registered in a formal database, but the protocol was documented prior to data extraction.

### 2.2 Search Strategy

A systematic literature search was conducted across three databases—Web of Science (WoS) Core Collection, Scopus, and PubMed—on 15 May 2026 (WoS) and 11 July 2026 (Scopus, PubMed). The same search logic was applied across all three databases, adapted to each platform's syntax:

(‘poultry’ OR ‘broiler’ OR ‘layer’ OR ‘chicken’) AND (‘protein source’ OR ‘soybean meal’ OR ‘fishmeal’ OR ‘alternative protein’ OR ‘insect meal’ OR ‘DDGS’ OR ‘microalgae’ OR ‘single‐cell protein’) AND (‘sustainability’ OR ‘nutrition’ OR ‘feed’)

restricted to 1999–2026. In PubMed, results were further restricted to non‐human animal species and to journal articles/reviews. Searches yielded 122 records from WoS, 5920 from Scopus, and 1827 from PubMed. Reviewer 2 raised the question of why the original submission relied on WoS alone; this revision addresses that comment directly by adding Scopus and PubMed with matched search logic (see Section 2.4 and Figure 1).

**Figure 1:**
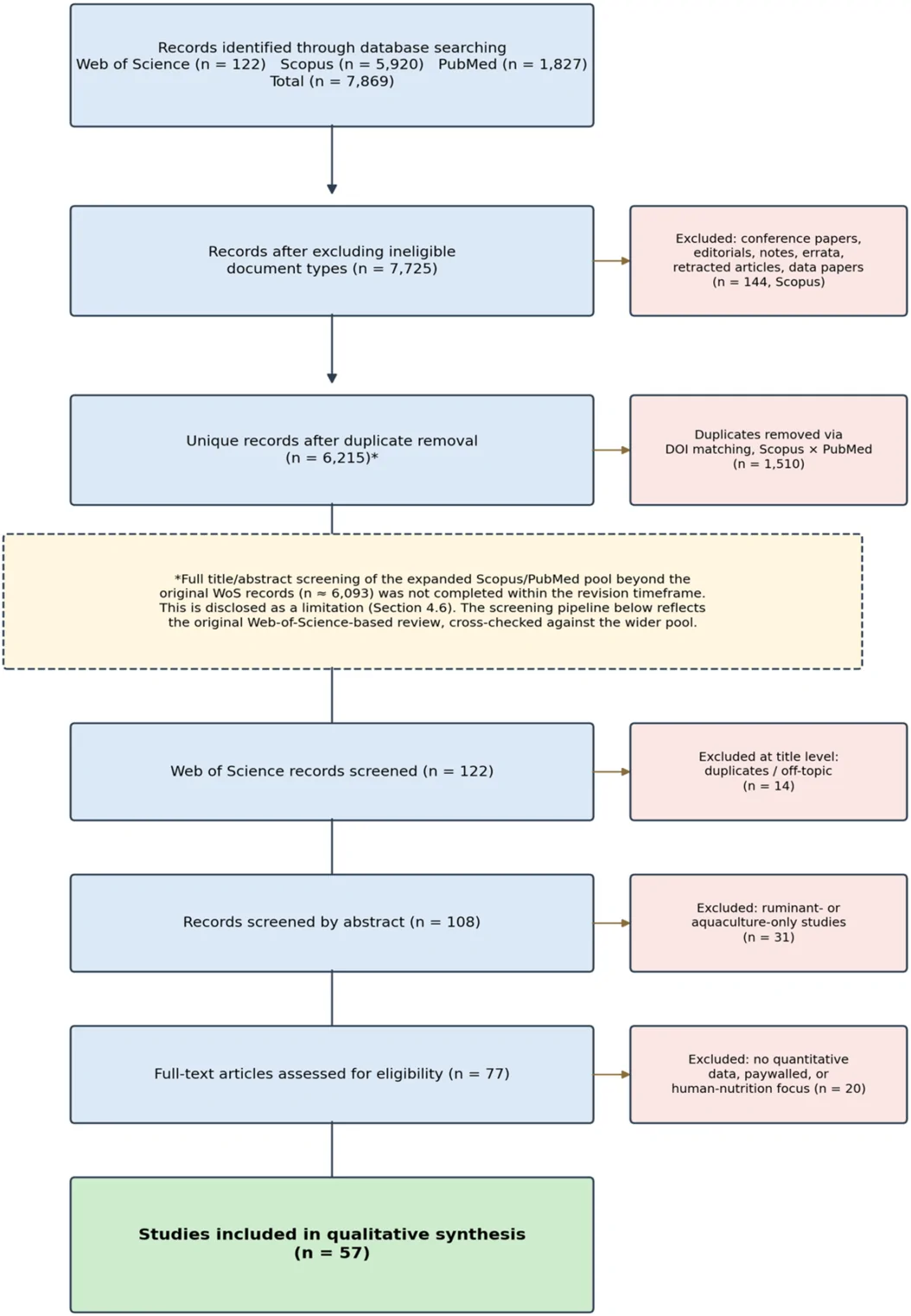
PRISMA 2020 flow diagram of the literature identification, screening, and inclusion process. Records identified across Web of Science, Scopus, and PubMed; qualitative synthesis based on the WoS‐derived screening pipeline (57 included studies), per Section 2.4. [Color figure can be viewed at wileyonlinelibrary.com]

### 2.3 Inclusion and Exclusion Criteria

Records were screened against the PRISMA criteria presented in Table 1. Full‐text articles were assessed for eligibility by title, abstract, and full‐text review. Studies were included if they reported empirical or quantitative data on protein sources for poultry nutrition, sustainability analyses using established life cycle assessment (LCA) or related metrics, or FAOSTAT‐integrated analyses of global supply and demand trends.

**Table 1:** PRISMA inclusion and exclusion criteria applied in the literature search.

Criterion	Inclusion	Exclusion
Study type	Peer‐reviewed journal articles, review articles, systematic reviews, meta‐analyses, book chapters	Conference abstracts, editorials, opinion pieces, grey literature without peer review
Language	English	Non‐English publications
Publication year	1999–2026	Before 1999
Topic relevance	Poultry feed protein sources, alternatives to soybean/fishmeal, sustainability, FAOSTAT data on poultry/soybeans	Studies on ruminants only, aquaculture exclusively, human nutrition, or unrelated feed additives
Data availability	Full‐text accessible; quantitative or qualitative data on protein source performance or sustainability	Abstract‐only publications; no measurable outcomes reported
Species focus	Broilers, layers, ducks, turkeys, quail, and other poultry species	Exclusively ruminant, pet animal, or aquaculture studies

#### 2.3.1 Quality Appraisal and Screening Process

Title/abstract and full‐text screening for the WoS pipeline (Figure 1) was conducted by the corresponding author. A formal risk‐of‐bias or methodological quality‐appraisal tool (e.g., AMSTAR‐2, ROBIS) was not applied, given the heterogeneity of study designs (feeding trials, LCA studies, and descriptive production analyses) included in this narrative‐style synthesis; instead, eligibility was restricted to peer‐reviewed sources reporting measurable outcomes (Table 1). We acknowledge single‐reviewer screening and the absence of a formal quality‐appraisal tool as limitations of this review (see Section 4.6), and recommend dual independent screening and a structured appraisal tool such as AMSTAR‐2 for future updates of this literature.

Paywalled articles identified during the initial search were retrieved using institutional access via the Hungarian University of Agriculture and Life Sciences (MATE); the exclusion of a small residual set of publications without full‐text access (via MATE or otherwise) is acknowledged as a minor, largely mitigated limitation rather than a systematic source of selection bias.

### 2.4 PRISMA Flow Diagram

From the 7869 records identified across WoS, Scopus, and PubMed, 144 Scopus records were excluded as ineligible document types (conference papers, editorials, notes, errata, retracted articles, data papers), leaving 7725. Duplicate removal via DOI matching between Scopus and PubMed identified 1510 confirmed duplicates, yielding 6215 unique candidate records (WoS records could not be DOI‐matched against the other two databases at the individual‐record level, so this figure carries a small unquantified residual duplication risk). Consistent with the original review, the qualitative synthesis reported here is based on the WoS‐driven screening pipeline: 14 records were excluded as duplicates or clearly off‐topic at the title level (108 remaining), 31 were excluded after abstract screening as ruminant‐ or aquaculture‐only studies (77 remaining), and a further 20 were excluded after full‐text review for lacking quantitative data, being paywalled, or focusing exclusively on human nutrition. Fifty‐seven studies were ultimately included in the qualitative synthesis. Full title/abstract and full‐text screening of the wider Scopus/PubMed pool (approximately 6093 additional unique records) was not completed within the revision timeframe; a targeted spot‐check of high‐relevance titles in this pool did not surface studies that appeared to materially change the synthesis, but we present this as a supplementary coverage check rather than a completed second screening pass (see Limitations, Section 4.6). No meta‐analysis was performed due to heterogeneity of outcome measures across studies.

### 2.5 FAOSTAT Data Analysis

FAOSTAT data sets were downloaded in May 2026 for: (1) Crops and Livestock Products domain—chicken stocks, chicken meat production, and soybean production (2000–2024); and (2) Crops and Livestock Trade domain—soybean import and export quantities (2000–2024). Data were aggregated across all countries to derive global totals for each year. The derived summary statistics are presented in Tables 2, 3, 4, 5 and used throughout this review to contextualise literature findings.

**Table 2:** Global chicken stocks and meat production trends (2000–2024).

Year	Chicken stocks (M head)	Meat production (kt)	Meat/bird (g)	Growth versus prior period (%)
2000	14,817	69,885	176	—
2005	18,247	80,143	219	+24.4
2010	25,412	99,444	250	+14.2
2015	26,681	118,180	301	+20.4
2018	29,779	129,326	335	+11.4
2020	31,252	133,621	344	+2.7
2022	31,412	137,243	352	+2.3
2024	32,223	144,000	360 (est.)	+1.9

*Note:* Stocks in millions of head; meat production in thousand tonnes (kt).

*Source:* FAOSTAT (2026).

**Table 3:** Top 10 chicken‐producing countries by stock and meat production (2022).

Rank	Country	Stocks (M head)	Meat Prod. (kt)	Region
1	China (mainland)	5194	15,800	Asia–Pacific
2	Indonesia	3482	3905	Asia–Pacific
3	USA	1532	22,012	North America
4	Pakistan	1725	1955	Asia–Pacific
5	Brazil	1569	15,234	South America
6	Iran	1036	2810	Middle East
7	India	837	4400	Asia–Pacific
8	Mexico	610	3650	North America
9	Russia	488	5220	Europe
10	Bangladesh	366	680	Asia–Pacific

*Source:* FAOSTAT (2026).

**Table 4:** Global soybean production, imports, and exports (2000–2024).

Year	Production (Mt)	Imports (Mt)	Exports (Mt)	Growth (%)
2000	161.1	60.3	51.2	+–
2005	214.5	81.2	62.4	+33.1
2010	280.2	153.4	97.5	+30.6
2015	335.1	215.5	131.2	+19.6
2018	363.6	244.0	152.7	+8.5
2020	377.0	270.8	173.4	+3.7
2022	376.0	249.9	157.7	–0.3
2024	418.3	284.0	178.7	+11.3

*Note:* Values in million tonnes (Mt).

*Source:* FAOSTAT (2026).

**Table 5:** Top 10 soybean‐producing countries (2022).

Rank	Country	Production (Mt)	Export share (%)	Region
1	Brazil	121.3	~86	South America
2	USA	116.2	~75	North America
3	Argentina	43.9	~6	South America
4	China	20.3	~0	Asia–Pacific
5	India	15.0	~2	Asia–Pacific
6	Canada	6.5	~56	North America
7	Russia	6.0	~8	Europe
8	Paraguay	5.2	~70	South America
9	Bolivia	3.5	~50	South America
10	Ukraine	3.1	~40	Europe

*Note:* Production in million tonnes (Mt); export share is approximate.

*Source:* FAOSTAT (2026).

## 3 Results

### 3.1 Global Poultry Production Trends (FAOSTAT, 2000–2024)

Global chicken stocks grew from 14.8 billion head in 2000 to 32.2 billion head in 2024, representing an increase of 117% over 24 years (Table 2; Figure 2). Growth was most pronounced in Asia–Pacific, particularly China, Indonesia, Pakistan, and India (Table 3), though significant expansion also occurred in Brazil and the United States. The most rapid period of growth occurred between 2015 and 2017, during which global stocks increased by approximately 3.1 billion head.

**Figure 2:**
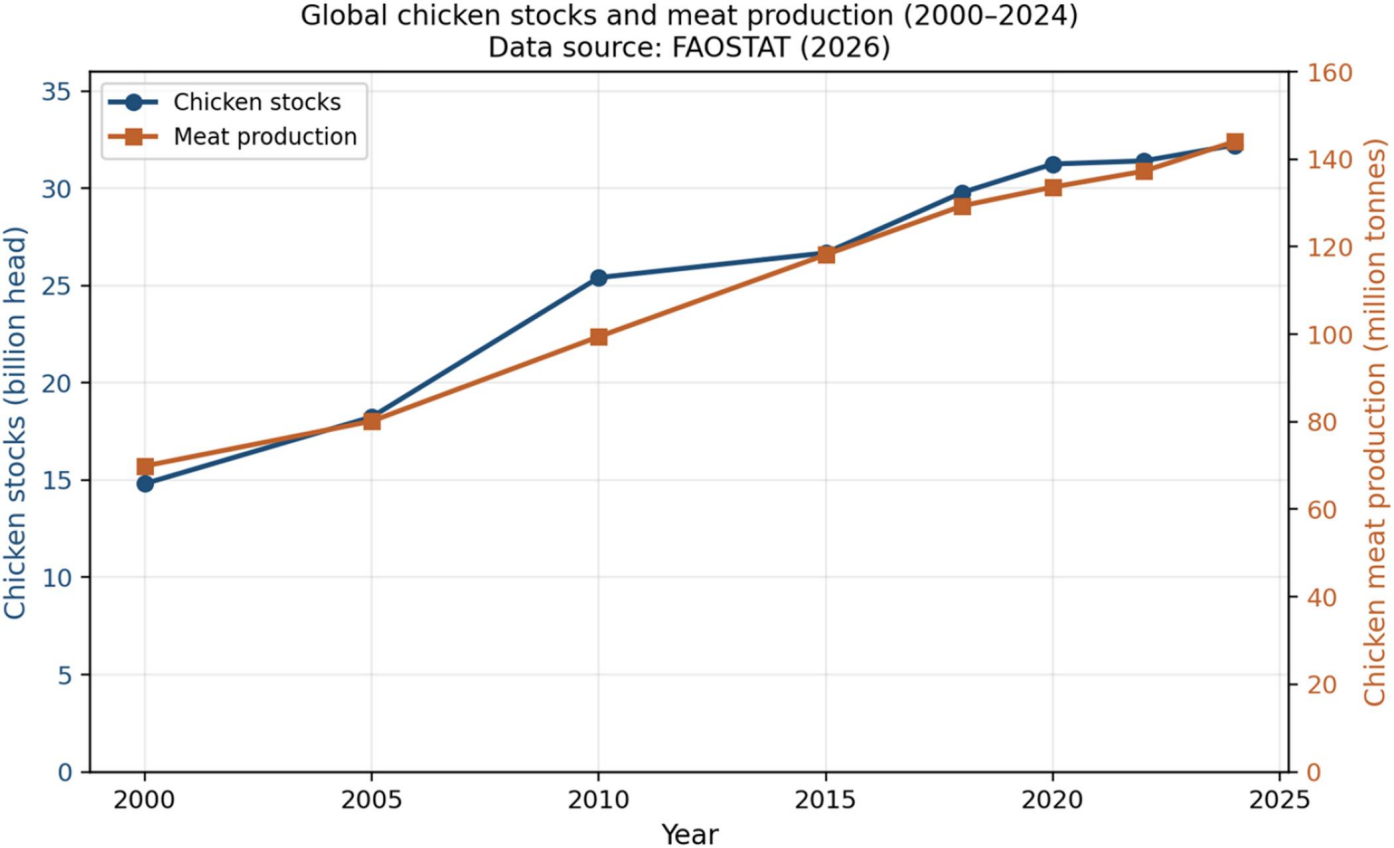
Global chicken stocks and chicken meat production (2000–2024). Left axis = chicken stocks (billion head, blue); right axis = meat production (Mt, orange). *Source:* FAOSTAT (2026). [Color figure can be viewed at wileyonlinelibrary.com]

Global chicken meat production followed a similar trajectory, rising from 69.9 Mt in 2000 to 144.0 Mt in 2024. Productivity gains—reflected in the increasing meat yield per bird—indicate that breeding, nutritional improvements, and management practices have amplified the absolute volume of meat generated per unit of bird population. The average carcass weight equivalent increased from approximately 176 g/bird in 2000 to 360 g/bird by 2024, reflecting the dominance of fast‐growing broiler breeds in commercial production. These per‐bird figures are derived by dividing FAOSTAT total meat production by total chicken stock for each year and are not drawn from a separate literature source.

### 3.2 Global Soybean Production and Trade Trends (FAOSTAT, 2000–2024)

Soybean is the single most important protein‐meal crop globally, providing the principal raw material for SBM used in commercial poultry diets. Global soybean production grew from 161.1 Mt in 2000 to 418.3 Mt in 2024, a 160% increase (Table 4 and Figure 3). The expansion in production has been concentrated in South America, particularly Brazil (121.3 Mt in 2022) and Argentina (43.9 Mt in 2022), alongside sustained production in the United States (116.2 Mt in 2022) (Table 5).

**Figure 3:**
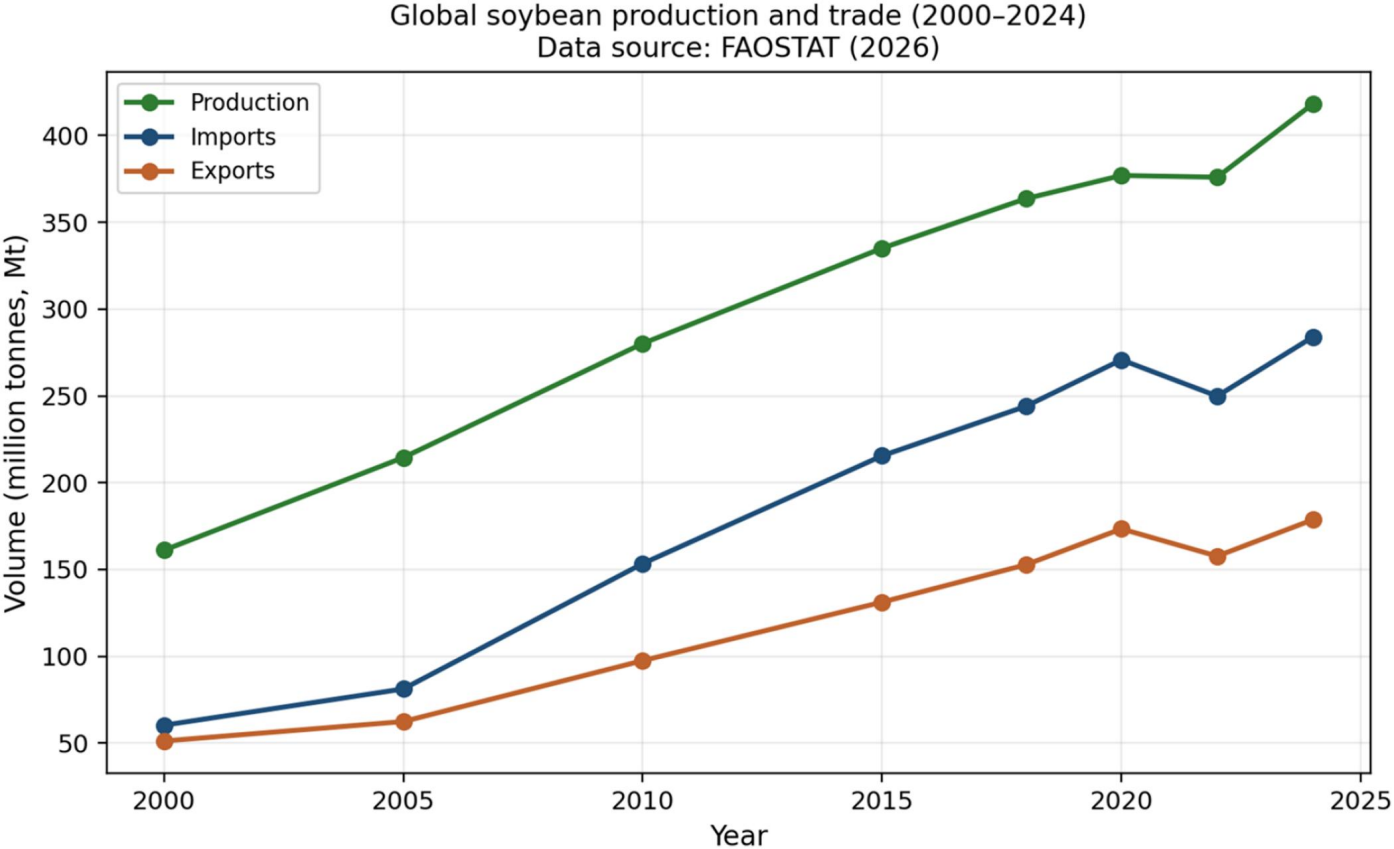
Global soybean production, import quantities, and export quantities (2000–2024). *Source:* FAOSTAT (2026). [Color figure can be viewed at wileyonlinelibrary.com]

Global soybean imports more than doubled from 60.3 Mt in 2000 to 284.0 Mt in 2024, with China accounting for the dominant share of import growth as its domestic livestock sector expanded. Soybean exports similarly grew from 51.2 Mt in 2000 to 178.7 Mt in 2024, illustrating the highly globalised and concentrated nature of this supply chain. The geographic concentration of production in two South American countries and the United States creates significant systemic risk from climate variability, geopolitical disruptions, and infrastructure challenges (Nyameasem et al. 2026).

### 3.3 Conventional Protein Sources in Poultry Nutrition

#### 3.3.1 SBM

SBM remains the cornerstone of commercial poultry diets worldwide, providing 44%–48% CP with a well‐balanced amino acid profile, comparatively high digestibility, and consistent availability (Toomer et al. 2024). Full‐fat soybeans offer additional metabolisable energy and are used where oil extraction infrastructure is limited (Toomer et al. 2024). However, raw soybeans contain anti‐nutritional factors (ANFs) including trypsin inhibitors, lectins, and phytate, necessitating heat treatment prior to use (Ravindran 2015). Globally, SBM consumption is projected to continue rising in line with poultry and swine expansion, maintaining sustained pressure on land and water resources, particularly in soybean‐producing biomes (Nyameasem et al. 2026).

#### 3.3.2 Fishmeal

Fishmeal provides 60%–72% CP with an excellent essential amino acid profile and high bioavailability, making it a premium ingredient in starter and breeder poultry diets (Tacon and Metian 2009). Its unsustainability stems from specific, quantifiable pressures rather than fishmeal scarcity alone: fishmeal is predominantly manufactured from small pelagic ‘reduction fisheries’ (e.g., anchoveta, sardine, and menhaden), many of which are fished at or beyond maximum sustainable yield, and reduction‐fishery catches are additionally exposed to illegal, unreported, and unregulated (IUU) fishing pressure that undermines stock‐assessment reliability (Tacon and Metian 2009). Combined with rising aquaculture demand for the same raw material, these factors make large‐scale, long‐term expansion of fishmeal supply for poultry diets structurally unsustainable. Research has consistently demonstrated that partial or total replacement of fishmeal with processed insect meals, SCPs, or co‐products is technically feasible without compromising performance (Dabbou et al. 2021; Adesogan et al. 2021).

#### 3.3.3 Poultry By‐Product Meal and Feather Meal

Poultry by‐product meal (PBM, ~60%–65% CP) and hydrolysed feather meal (HFM, ~80%–85% CP) are valuable co‐products of the poultry processing industry, representing circular‐economy solutions within the sector. Sauvant et al. (2002) reported acceptable digestibility for PBM in monogastric diets, while HFM digestibility is substantially improved by autoclaving or enzyme supplementation. García‐Ruiz and Durán‐Meléndez (2022) highlighted the potential of these ingredients to reduce net protein demand in feed formulation, provided consistent quality control is maintained (Table 6).

**Table 6:** Comparative nutritional and sustainability profiles of major poultry feed protein sources.

Protein source	CP (% DM)	AA profile	Cost	Availability	Key limitations
Soybean meal	44–48	High	Low–Medium	High	Land use, deforestation
Fishmeal	60–72	Very high	High	Medium	Overfishing, wild stock depletion
Black soldier fly larvae	40–55	High	Low	Medium–High	Scale‐up, regulation
Mealworm (T. molitor)	45–60	High	Low	Medium	Energy‐intensive rearing
Single‐cell protein	50–70	High	Low	Low–Medium	Substrate cost, scale
Canola/rapeseed meal	35–38	Medium	Low	High	Glucosinolates if unprocessed
DDGS	27–30	Medium	Low	High	Mycotoxin risk, variable quality
Microalgae (spirulina)	55–65	High	Low	Low	Production cost
Feather Meal (hydrolysed)	80–85	Low–Medium	Very Low	Medium	Low digestibility if unprocessed
Poultry by‐product meal	58–65	Medium–High	Low	Medium	Biosafety, variability

Abbreviations: AA = amino acid profile quality; CP = crude protein; DM = dry matter.

### 3.4 Alternative and Emerging Protein Sources

#### 3.4.1 Insect Meals

Black soldier fly larvae (BSFL, Hermetia illucens) have been the most extensively studied insect protein source for poultry over the past decade. Their CP content ranges from 40% to 55% (dry matter basis). As in most poultry feedstuffs, methionine is generally the first‐limiting amino acid in insect meal, with cystine contributing to total sulphur amino acid supply rather than acting as an independent limiting factor. Reported methionine and cystine levels are generally somewhat lower than fishmeal on a metabolisable basis, though under optimised rearing substrates some formulations approach comparable levels; dietary balancing or targeted amino acid supplementation is therefore recommended when BSFL meal is included at higher inclusion rates (Zaid et al. 2023; Veldkamp et al. 2022). LCAs consistently show lower land and water requirements than SBM on a protein‐equivalent basis, particularly when BSFL are reared on organic waste streams (Smetana et al. 2019). Studies reviewed confirm partial SBM replacement at 15%–25% inclusion without significant adverse effects on broiler growth performance, feed conversion ratio (FCR), or carcass quality (Dabbou et al. 2021; Zaid et al. 2023); reported outcomes are not fully uniform across studies, however, and inclusion above this range has in some trials been associated with reduced FCR, underscoring that substrate composition and inclusion level both materially affect outcomes rather than BSFL performing as a uniform drop‐in replacement.

Yellow mealworm (T. molitor) similarly provides 45%–60% CP and a favourable essential amino acid profile (Arioli et al. 2020). Nguyen et al. (2023) and Swiatkiewicz et al. (2020) documented positive palatability and performance outcomes, and consumer acceptance for poultry products from mealworm‐fed birds has been reported as moderate to high when information is provided (Lähteenmäki‐Uutela et al. 2021). Regulation in the European Union has progressively expanded to allow processed insect proteins in poultry and pig feed as of 2021 under Commission Regulation (EU) 2021/1372, catalysing commercial scale‐up (European Commission 2021).

Other insect species studied include the housefly (Musca domestica) larvae, mopane worm (Gonimbrasia belina) for quail (Dreassi et al. 2021), and Mediterranean fruit fly rearing residues (reviewed by Bellezza Oddon et al. 2025). Each offers contextually relevant solutions, especially in regions where specific insect species are culturally and regulatorily accepted.

#### 3.4.2 SCPs

SCPs include yeast (Saccharomyces cerevisiae, Candida utilis), bacteria (Methylococcus capsulatus), and fungi (Fusarium venenatum—marketed as Quorn mycoprotein). CP content typically ranges 50%–70% DM, with a favourable essential amino acid balance. Burton et al. (2013) demonstrated that a yeast protein concentrate derived as a bioethanol co‐product could partially replace SBM in broiler diets at inclusion levels of up to 10%, maintaining comparable growth performance and FCR. García‐Ruiz and Durán‐Meléndez (2022) provide a roadmap of SCP research directions, noting that the principal barriers are substrate cost and fermentation scale‐up rather than intrinsic nutritional value.

#### 3.4.3 Microalgae

Microalgae—particularly Spirulina (Arthrospira platensis) and Chlorella vulgaris—contain 55%–65% CP with high bioavailability and unique profiles of essential fatty acids, pigments, and bioactive compounds (Yildirim et al. 2017; Chiofalo et al. 2024). Their inclusion in poultry diets at 2%–5% levels has been shown to improve egg yolk colouration, meat oxidative stability, and immune competence. However, production costs remain substantially higher than SBM, limiting economic viability at commercial scale. Aquaculture‐derived microalgae cultivation systems may offer a pathway to cost reduction through nutrient recycling and integration with wastewater treatment.

#### 3.4.4 Co‐Products: DDGS, Canola Meal, and Legumes

DDGS contain 27%–30% CP with elevated B‐vitamins and are produced in large volumes as a co‐product of bioethanol manufacture (Lim et al. 2008; Leiva et al. 2022). Their inclusion in poultry diets at 5%–15% is well‐tolerated in broilers and layers, though high fibre content and variable energy values necessitate careful formulation, and the risk of mycotoxin contamination requires monitoring (Nunes et al. 2021). Canola meal (35%–38% CP) has been evaluated as a cost‐effective partial SBM substitute in regions where canola is produced; because raw canola meal contains glucosinolates that can depress feed intake and thyroid function at high inclusion, appropriate processing (heat treatment, low‐glucosinolate cultivars) is required to realise the performance parity demonstrated in recent broiler trials (Murugesan et al. 2025). Legumes including peas, beans, and lupins are similarly promising as locally sourced protein supplements, though raw legume seeds carry their own ANFs (trypsin inhibitors, tannins) that must be addressed through processing; Michiels et al. (2020) reported equivalent broiler performance to SBM‐based diets once these were accounted for in European trials.

#### 3.4.5 Unconventional and Novel Sources

A range of additional protein sources have been assessed in the included literature, reflecting the breadth of global research. Acacia leaf meal provides moderate CP (18%–22%) and has been reviewed as a supplementary protein and functional additive for poultry in sub‐Saharan Africa (Zaid et al. 2022). Giant squid (Dosidicus gigas) meal has been assessed as an n‐3 fatty acid‐enriched substitute for fishmeal in broiler diets, demonstrating performance equivalence to SBM at moderate inclusion (Oso et al. 2022). Natural rubber serum protein, HFM, pineapple waste meal, and Azolla pinnata have each been explored in tropical and subtropical contexts as low‐cost local protein sources (Zannou and Opiyo 2025).

## 4 Discussion

### 4.1 The Soybean–Poultry Nexus and Its Sustainability Limits

The FAOSTAT data synthesised in this review illustrate an historically unprecedented co‐expansion of poultry production and soybean cultivation. Between 2000 and 2024, global chicken meat output nearly doubled while global soybean production nearly tripled, and soybean imports grew more than fourfold. This trajectory has delivered real benefits—affordable poultry protein accessible to low‐income consumers worldwide—but has come at mounting ecological cost. The concentration of soybean production in Brazil and Argentina, where expansion frequently occurs at the expense of Cerrado and Amazon biome habitats, means that the global poultry supply chain carries a measurable deforestation risk (Nyameasem et al. 2026; Wiedemann et al. 2013).

Climate volatility in key South American producing regions imposes supply disruptions and price shocks that ripple across the global poultry sector, disproportionately affecting lower‐income producing and consuming countries. Diversifying the protein ingredient base is therefore not merely an environmental objective but a strategic resilience imperative for the global feed industry.

### 4.2 Insects as Transformative Protein Sources

The reviewed literature strongly supports BSFL as the most technically mature and commercially scalable insect‐derived protein currently available for poultry feeding. The convergence of regulatory acceptance (Commission Regulation [EU] 2021/1372 permitting insect proteins in poultry and pig feed), an increasing number of commercial‐scale facilities, and performance data across broiler, layer, and quail species positions BSFL as the foremost near‐term alternative to SBM in formulations where it is economically competitive (Nguyen et al. 2023; Veldkamp et al. 2022; Van Huis et al. 2013). The circular‐economy dimension—rearing BSFL on pre‐consumer organic waste streams—provides compounded environmental benefits by diverting organic waste from landfill, producing a nutrient‐dense meal, and generating frass as a biofertiliser.

Nevertheless, critical challenges remain. As noted in Section 3.4.1, methionine levels in BSFL are generally lower than in fishmeal, necessitating supplementation or dietary balancing in most formulations; reports of comparable or superior amino acid profiles in the literature appear specific to particular rearing substrates and should not be generalised as a default BSFL characteristic. Lipid composition varies considerably with substrate composition, which can affect meat quality parameters in broilers and egg fatty acid profiles in layers (Dabbou et al. 2021). Regulatory frameworks outside the European Union, particularly in key growth markets in Asia and Africa, remain fragmented, creating barriers to international trade in insect‐derived ingredients.

### 4.3 Phytase and Feed Enzyme Integration

Across the reviewed literature, the integration of exogenous enzymes—particularly phytases, proteases, and carbohydrases—was identified as an enabling technology for maximising the nutritional value of alternative protein sources. Phytase supplementation is of particular importance, as high‐phytate ingredients such as SBM, canola meal, and DDGS bind phosphorus and reduce the bioavailability of other minerals and amino acids (Ravindran 2015). Incorporating appropriate enzyme cocktails into diets based on alternative protein sources can substantially improve their competitiveness with conventional SBM‐based formulations.

### 4.4 Environmental and Sustainability Dimensions

LCA studies included in this review consistently show that alternative protein sources—particularly insects and SCP—generate lower greenhouse gas emissions, require less agricultural land, and consume less freshwater per unit of protein produced compared with SBM and especially fishmeal (Smetana et al. 2019; Wiedemann et al. 2013). For poultry producers operating in markets with mandatory environmental disclosure, carbon border adjustment mechanisms, or sustainability certification requirements, transitioning to alternative proteins represents not only an ecological benefit but a competitive advantage.

These qualitative patterns are corroborated by quantitative findings in the reviewed literature. Wiedemann et al. (2013) reported that substituting a share of soybean‐based protein with alternative crop‐based ingredients in Australian broiler and egg‐production systems reduced global warming potential by more than 10% relative to a soy‐based baseline diet. For BSFL meal specifically, reported global warming potential values in the literature span an approximately fourfold range depending on rearing substrate, energy source, and system‐boundary assumptions, with feed‐substrate production and energy inputs for heating, ventilation, and processing consistently identified as the dominant contributors to the overall footprint (Smetana et al. 2019). This variability reinforces that BSFL's environmental advantage over conventional protein sources cannot be assumed uniformly and depends on the specific production system evaluated.

The environmental performance of alternative proteins, however, is highly context‐dependent. Where SCP production relies on fossil fuel‐derived methane as a substrate, net GHG benefits may be minimal. Where BSFL are reared on regulated organic waste in energy‐intensive facilities, energy consumption may dominate the life cycle footprint. A nuanced, site‐specific approach to sustainability assessment is essential, integrating local energy mix, substrate availability, transport logistics, and land‐use context.

### 4.5 Consumer Acceptance and Regulatory Considerations

Consumer acceptance of poultry products derived from insects, algae, or novel co‐products is an evolving area of research. The included literature indicates that when consumers are provided with factual information about the environmental and nutritional benefits of alternative feeds, acceptance is substantially higher than when information is withheld (Lähteenmäki‐Uutela et al. 2021). Regulatory harmonisation at the international level—particularly alignment of CODEX Alimentarius standards with national food safety legislation—will be critical for enabling market access and international trade in novel feed ingredients.

### 4.6 Limitations

This review has several limitations that should inform interpretation of its findings. First, screening was conducted by a single reviewer without a second independent screener, and no formal risk‐of‐bias or quality‐appraisal tool was applied (Section 2.3.1); both are recommended additions for future updates. Second, while paywalled articles were retrieved where possible via institutional access (MATE), a small residual set of publications may remain inaccessible through any channel available to the authors; this is considered a minor rather than systematic limitation. Third, while the search was expanded from a single database (WoS) to three databases (WoS, Scopus, PubMed) in response to peer review, full title/abstract and full‐text screening of the substantially larger Scopus/PubMed record pool (approximately 6093 additional unique records beyond the original WoS set) was not completed within the revision timeframe; the qualitative synthesis therefore remains grounded in the original 57 WoS‐derived studies, cross‐checked against the wider pool only via a targeted spot‐check rather than a full second screening pass. Fourth, heterogeneity in outcome measures across the included studies precluded meta‐analysis, limiting this review to qualitative rather than quantitative synthesis. Future work should prioritise a complete multi‐database re‐screen, dual independent reviewers, and a formal quality‐appraisal instrument.

## 5 Conclusions and Recommendations

This review, integrating FAOSTAT production and trade data with evidence from 57 peer‐reviewed publications identified via a WoS‐led, multi‐database‐informed search, documents the substantial growth of global poultry production between 2000 and 2024 alongside a concurrent expansion of soybean cultivation that is approaching ecological and geopolitical sustainability limits. The literature supports the technical feasibility of partially or fully replacing SBM and fishmeal with a diverse range of alternative protein sources, including BSFL, mealworms, SCP, microalgae, DDGS, canola meal, and locally sourced plant and animal by‐products, though performance outcomes depend materially on substrate, inclusion level, and processing.

Based on the evidence reviewed, we recommend that:

- Research institutions prioritise long‐term feeding trials with alternative protein combinations rather than single‐ingredient substitution studies, to better reflect commercial diet formulation practice.
- Feed industry actors invest in pilot‐ and commercial‐scale processing infrastructure for insect meal and SCP to reduce unit costs and ensure consistent quality standards.
- Regulatory bodies accelerate harmonised, evidence‐based standards for novel feed ingredients, particularly around food safety, labelling, and cross‐border trade.
- Sustainability assessments of poultry supply chains incorporate dynamic FAOSTAT data to ensure environmental claims remain grounded in current, regionally specific evidence.
- Future systematic updates of this literature complete a full multi‐database screen, apply dual independent reviewers and a formal quality‐appraisal tool, and develop transparent consumer communication on the provenance of alternative‐protein‐fed poultry products.

The transition from a soybean‐centric global poultry protein supply to a diversified, regionally adapted, circular‐economy‐oriented system is both necessary and achievable; coordinated action across research, policy, industry, and civil society will determine its pace and equity.

## Ethics Statement

This manuscript is a systematic/data‐based review study using publicly available FAOSTAT data and previously published literature. No live animals were used directly in this study, and therefore ethical approval or animal welfare approval was not required.

## Conflicts of Interest

The author declares no conflicts of interest.

## Data Availability

Data sharing not applicable to this article as no data sets were generated or analysed during the current study.
